# Excavation of Genes Related to the Mining of Growth, Development, and Meat Quality of Two Crossbred Sheep Populations Based on Comparative Transcriptomes

**DOI:** 10.3390/ani11061492

**Published:** 2021-05-21

**Authors:** Jinping Shi, Xueying Wang, Yali Song, Ting Liu, Shuru Cheng, Quanwei Zhang

**Affiliations:** 1 College of Animal Science and Technology, Gansu Agricultural University, Lanzhou 730070, China; shijinpingyunfei@163.com (J.S.); Songyl1015@163.com (Y.S.); liuting0628@163.com (T.L.); 2 College of Veterinary Medicine, Gansu Agricultural University, Lanzhou 730070, China; zqwfree826@163.com; 3 College of Life Science and Biotechnology, Gansu Agricultural University, Lanzhou 730070, China

**Keywords:** sheep, crossbreed, transcriptome, muscle, growth, development, meat quality, genetics

## Abstract

**Simple Summary:**

In this study, we measured the performance parameters of two crossbred sheep breeds, using Masson staining of the muscle tissue, and using the Illumina high-throughput sequencing platform to determine the differentially expressed genes (DEGs) in Dorper (DP) × Small-tailed Han (STH) sheep and Mongolia (MG) × Small-tailed Han sheep (STH). New transcripts of the muscle transcriptome were examined for the first time. DP × STH sheep were superior to MG × STH sheep in terms of meat quality and muscle morphology. In addition, 13 DEGs were found to play important roles in growth, development, and meat quality. The findings of this work may provide valuable resources for future research on muscle development in sheep.

**Abstract:**

Crossbreeding can improve production performance and meat quality in sheep. The objective of this study was to look for genes related to sheep growth, development, and muscle. In this study, Dorper (DP) × Small Tailed Han (STH) sheep and Mongolia (MG) × Small-tailed Han (STH) sheep were used to estimate the productive performance and meat quality in a crossbreed. Subsequently, transcriptome analysis and bioinformatic analysis were performed on the *Longissimus dorsi* muscles of DP × STH and MG × STH sheep to identify differentially expressed genes (DEGs) related to growth, development, and meat quality. The presence of DEGs was confirmed by real-time PCR (qPCR). Productive performance and meat quality of the DP × STH sheep were better than the MG × STH sheep. Compared to DP × STH, a total of 1445 DEGs were identified in MG × STH sheep (1026 DEG were up-regulated and 419 DEG were down-regulated). Of these, 38 DEGs were related to growth, 161 to development, and 43 to muscle. In addition, 13 co-expressed genes (*FGFRL1*, *SIX1*, *PLCB1*, *CRYAB*, *MYL2*, *ADIPOQ*, *GPX1*, *PPARD*, *GPC1*, *CDC42*, *LOC101106246*, *IGF1*, and *LARGE*) were identified. The expression of DEGs was consistent with the comparative transcriptome analysis. This work provides genetics resources for future research on muscle development in sheep.

## 1. Introduction

China has a large animal husbandry industry with abundant sheep germplasm resources, accounting for 10% of the world’s approximately 700 sheep breeds [1]. Small-tailed Han sheep (STH) are renowned for their strong adaptability, fecundity, stress resistance, and tolerance of low-quality feed. They are widely bred in northwest China, where the natural environment is barren, and the grasslands are limited. Compared with foreign sheep breeds, Small-tailed Han sheep have obvious deficiencies in productive performance and meat quality. Significant effects have been achieved with breeding improvements in lactation and disease resistance [2]. Crossbreeding programs for traits such as growth, development, and meat quality in sheep are developing rapidly. In order to improve performance and meat quality in sheep while retaining resistance to stress, Small-tailed Han sheep are often used as the female parent to cross with sheep breeds from abroad with good productive performance [3]. Dorper sheep, native to South Africa, are a meat breed made by crossing South African black-headed Persian ewes as the female parent and British Dorset Horn sheep as the male parent. Dorper lambs grow fast, have a large weaning body, and have good meat quality. Mongolian sheep are one of the three major coarse-wool sheep breeds in China. They have the characteristics of strong viability, suitable for nomadism, cold tolerance and drought tolerance, and have good meat and fat performance. Excellent Dorper sheep and Mongolian sheep are often used as a male parent for crossbreeding. In northwest China, Dubo sheep and Mongolian sheep are crossed with Small-tailed Han sheep on a large scale, and the hybrid population accounts for about 30% of the total sheep.

In the past 20 years, RNA-sequencing technology has developed rapidly in the field of biology due to its powerful sequencing function [4], and it has come into its own for the study of differentially expressed genes (DEGs) and new transcript units in various organisms, including chickens [5], pigs [6], and cattle [7]. In recent years, transcriptome analysis of crossbred sheep and their ontologies have been widely studied. Transcriptome analysis of skeletal muscles in Duper sheep (DP) and STH sheep breeds has shown that there are many gene expression differences in muscle growth and development genes such as *ITGBL1*, *CPXM2*, *MRPL1*, *HPSE*, *SIGLEC14*, and *APOBEC3G* [8,9]. Some studies have analyzed the heat stress of Hu sheep through the transcriptome. Heat stress triggers complex changes in the expression of genes related to the hypothalamus, affecting the assembly and function of ribosomes through the calcium signaling pathway, which directly affects growth and development [10]. DEGs closely related to muscle growth, development, and muscle fibers (myosin, troponin, myoglobin) were found in the muscle transcriptome of DP and STH sheep [11]. Studies have found that 34 DEGs (including *ECM*, *IGFDP5*, and *TGFBR3*) are related to the development and differentiation of muscle cells, which is similar to the expression patterns observed in other animals [12]. Some studies have also identified muscle regulators, such as *MYOD1* and *MYOG*, as key mediators of skeletal muscle formation and they control muscle generation in the form of cross-coronal beams [13]. Comparative transcriptome analysis of the *Longissimus dorsi* tissue of Chinese Merino sheep (a crossbred sheep) and STH [14] indicated that myogenic regulatory factors (MRFs), ubiquitin-like modifier activating enzyme 1 (*GXP1*)*,* SH3 and cysteine-rich domain 3 (*STAC3*) and other DEGs play an important role in the process of muscle growth and development. Although transcriptome analysis of sheep, as described above, has been extensively studied, transcriptome analysis between crossbreeds has not been reported. Therefore, it is of significance to molecular breeding to understand the regulatory mechanisms of muscle growth, development, and meat quality among different crossbred sheep populations from the perspective of genetics, and to explore the regulatory genes. In this study, transcriptome comparative analysis was performed between crossbred DP and STH, and Mongolian sheep (MG) and STH sheep in order to identify the important genes affecting the productive performance and meat quality of the *Longissimus dorsi* tissue. This study provides new candidate genes for genetic and molecular studies of sheep muscle growth and a theoretical basis for improving sheep performance and lamb and mutton quality in future genetics.

## 2. Materials and Methods

### 2.1. Sample Preparation, Collection, and Measures of Productive Performance 

All samples were collected in strict accordance with the code of ethics approved by the Animal Welfare Committee of the College of Animal Science and Technology of Gansu Agricultural University (GSAU-AEW-2017-0308).

Samples of the *Longissimus dorsi* of sheep were taken. Samples were collected from Small-tail Han sheep (female), Dorper sheep (male), and Mongolian sheep (male), which are endemic to Zhangye City, Gansu Province, China. In this study, we chose lambs of 10 ± 1 d of age (DP × STH, *n* = 50) and (MG × STH, *n* = 50). The sheep were raised exclusively in a warm shed with regular feeds of a commercial ration, ad libitum access to water, and the same feeding and management conditions. All lambs were weaned at 2 months of age. At 6 months of age, the DP × STH and MG × STH sheep were slaughtered, and their productive performance and meat quality were determined [14]. Before slaughter, the live weight of the test animals after fasting for 24 h was measured. After slaughter, carcass weight, psoas area, slaughter rate, net meat rate, and other traits were measured and calculated [15,16,17]. A sample (2.5 cm thick) was taken for 24 h aging, and to measure the brightness L*, redness a* and yellowness b* using the NPPC colorimetric plate under the same conditions of room temperature (approximately 24 °C) and light conditions. Each sample was measured twice. The water loss rate and water holding capacity during cooking (as a percentage) were determined by the pressure method and cooking method. Muscle shear force was measured using a C-LM3B muscle shear force meter (Tenovo, China). At 45 min and 24 h after death, a PB-10 portable pH meter was used to measure the pH of the *Longissimus dorsi* (between the 12th and 13th ribs), and marbling was scored [18]. Transcriptome analysis was performed by randomly selecting tissue samples from each crossbred population (*n* = 6). Muscle tissue samples from the *Longissimus dorsi* were collected, immediately frozen in liquid nitrogen, and stored at −80 °C until RNA extraction was performed. In addition, the fresh *Longissimus dorsi* was fixed with 10% formaldehyde for 24–48 h, dehydrated routinely, and embedded in paraffin for subsequent Masson’s Trichrome Staining.

### 2.2. Masson Staining

Masson’s trichrome staining is based on the principle that the size of anionic dye molecules and the permeability of tissues are different. Collagen fibers are dyed blue with aniline blue; muscle fibers are dyed red with acid fuchsin and Ponceau; red blood cells are orange-red; and the nucleus is black and blue.

The *Longissimus dorsi* was stained using the Masson Trichrome Staining Solution Kit (Solarbio, Beijing, China), according to the manufacturer’s instructions. Briefly, the sections were dewaxed and stained in Weigert’s iron hematoxylin working solution for 5–10 min. This was followed by acidic ethanol differentiation solution, rinsing with distilled water for 1 min, Masson blue solution for 3–5 min, rinsing with distilled water for 1 min, and Ponceau red magenta stain for 5–10 min. Phosphomolybdic acid solution was used for 1–2 min, the weak acid working solution was washed for 1 min, and aniline blue stain was used for 1–2 min. After staining, the tissues were analyzed using Image-J (National Institute of Health) software.

### 2.3. Total RNA Preparation, cDNA Library, and Sequencing

Trizol reagent (TinaGen, Beijing, China) was used to extract total RNA from the *Longissimus dorsi*, DP × STH and MG × STH, with six samples each. The RNA samples with an RNA Integrity Number (*RIN*) value >8.5 were used for cDNA library construction. The Ribo Zero™ magnetic kit (Epicentre, San Diego, CA, USA) was used for enrichment and fragmentation. The mRNA was used as a template to synthesize cDNA. Using the QiaQuick-PCR kit (Qiagen, California, CA, USA), double-stranded cDNA was synthesized and purified to obtain the final library. After the completion of the library construction, fragments with a size of 300 bp were detected in order to ensure the quality of the library. After the library tests were qualified, different libraries were pooled using a flow cell, according to the requirements of the effective concentration and target offline data volume. After cBOT clustering, the Illumina HiSeq™ 2500 (Illumina, San Diego, CA, USA) high-throughput sequencing platform (HiSeq/MiSeq) was used for sequencing.

### 2.4. Transcriptome Assembly and DEG Identification

Strict filtering of sequenced data was performed, such that reads containing sequencing joints, reads with >10% unknown nucleotide (N) ratios, and low-quality reads with *Q* ≤ 20 were removed. High-quality, clean reads were obtained for subsequent analysis. After quality control, they were mapped to the sheep genome (*Oar. v3.1*) by Bowtie2 and TopHat2 (version 2.0.3.12) [19,20]. Reconstructed transcripts from multiple groups were incorporated into a complete set of transcripts using cufflinks and TopHat2 [21] for expression analysis. Gene abundance was quantified by RSEM and Bowtie [22]. Gene expression levels were normalized using the Per Kilobase (FPKM) method, which is directly used to compare gene expression differences per million map reads [23]. The R software package [10] was used to screen differentially expressed genes (DEGs) with the criteria: |log2FC| > 1 and FDR < 0.05. All of the DEGs were used for gene ontology (GO) enrichment analysis [24] and Kyoto Encyclopedia of Genes and Genomes (KEGG) pathway analysis [25,26]. The pathways with *Q* < 0.01 were considered significantly enriched. We focused on the GO terms that related to growth, development, and meat quality in the sheep breeds because these traits are the main factors that affect the performance of domesticated animals. Finally, the DEGs from these GO terms were selected as the target DEGs for further analysis.

### 2.5. Gene Behavior Network and Co-Expression Analysis Targeting DEGs

Based on the relationships among genes, proteins, and compounds in the KEGG database, the gene act network was constructed using Cytoscape software (v3.7.2). A co-expression network was constructed based on the standardized signal strength of DEGs selected from significant GO items and pathways. The Spearman’s correlation coefficient of candidate DEGs was calculated to construct a correlation coefficient matrix for further study and determine the significance of co-expressed mRNA.

### 2.6. Quantitative Real-Time PCR Assays for Target Genes

Trizol reagent (Invitrogen, California, CA, USA) was used to extract total RNA from *Longissimus dorsi* tissue (six copies each of DP × STH and MG × STH). According to the instructions of the TRANS reverse transcription kit (TransGen Biotech Inc., Beijing, China), complementary DNA (cDNA) was synthesized by using a BioTeke Thermo RT Kit (Bioteke, Beijing, China) following the manufacturer’s instructions. RNA (700 ng) was used as a template for synthesizing cDNA with a 20 μL reaction volume. Quantitative real-time polymerase chain reaction (qPCR) was applied to detect the expression levels of the target genes *FGFRL1*, *SIX1*, *PLCB1*, *CRYAB*, *MYL2*, *ADIPOQ*, *GPX1*, *PPARD*, *GPC1*, *CDC42*, *LOC101106246*, *IGF1*, and *LARGE* (qPCR primer sequences are detailed in Table S1). The qPCR was performed on an ABI7300 real-time system (ABI system, Foster City, CA, USA) using a 20 μL reaction volume including 1 μL cDNA. The SYBR premix Ex Taq™ II and specific primers were used for each reaction. The expression of the housekeeping gene, *beta-actin,* was used as an in-group control. A denaturation step was run for one cycle at 95 °C for 30 s. The annealing step was run for 40 cycles at 95 °C for 5 s and 60 °C for 31 s. All PCR reactions were performed in triplicate. The results were calculated using the 2^−ΔΔCT^ method.

### 2.7. Statistical Analysis 

Using SPSS version 22.0 (SPSS Inc., Chicago, IL, USA), an independent-samples *t*-test was performed on the production performance, muscle fiber area, diameter, and fluorescence quantitative data of the two hybrid sheep populations. The data were expressed as the mean ± standard deviation. PRISM 5.0 (GraphPad Software Inc., La Jolla, CA, USA) was used to draw statistical graphs. *p*-values less than 0.05 were considered statistically significant.

## 3. Results

### 3.1. Productive Performance and Meat Quality

**Figure 1:**
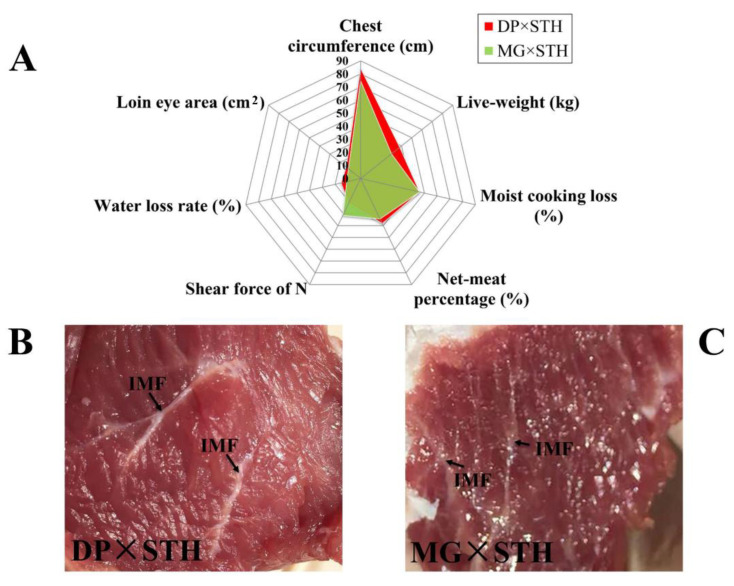
The significantly different indices related to performance and meat quality in the DP × STH and MG × STH sheep. (**A**) The significantly different indices of chest circumference, liveweight, carcass weight, loin eye area, and net-meat percentage were used for comparing the productive performance of DP × STH and MG × STH sheep. (**B**,**C**) The marbling and fat content of DP × STH and MG × STH sheep.

Productive performance and meat quality of DP × STH and MG × STH sheep were measured (Table S2). There were differences in chest circumference, live weight, carcass weight, net meat weight, net meat ratio, meat-to-bone ratio, slaughter rate, waist area, and back meat thickness (*p* < 0.01), but body height and body lengths were not different. The indices of the DP × STH group were higher than the MG × STH group (*p* < 0.01). The radar chart of several important parameters (chest circumference, live weight, moist cooking loss, net meat percentage, shear force (N), water loss rate, and loin eye area) illustrated the differences in productive performance and meat quality (Figure 1A). These results suggest that the performance of the DP × STH group was better than the MG × STH group. The marbling grade, redness (R*), and intramuscular fat (IMF) of the DP × STH group (Figure 1B) were higher than the MG × STH group (Figure 1C) by visual observation. 

### 3.2. Masson Staining of the Longissimus dorsi

**Figure 2:**
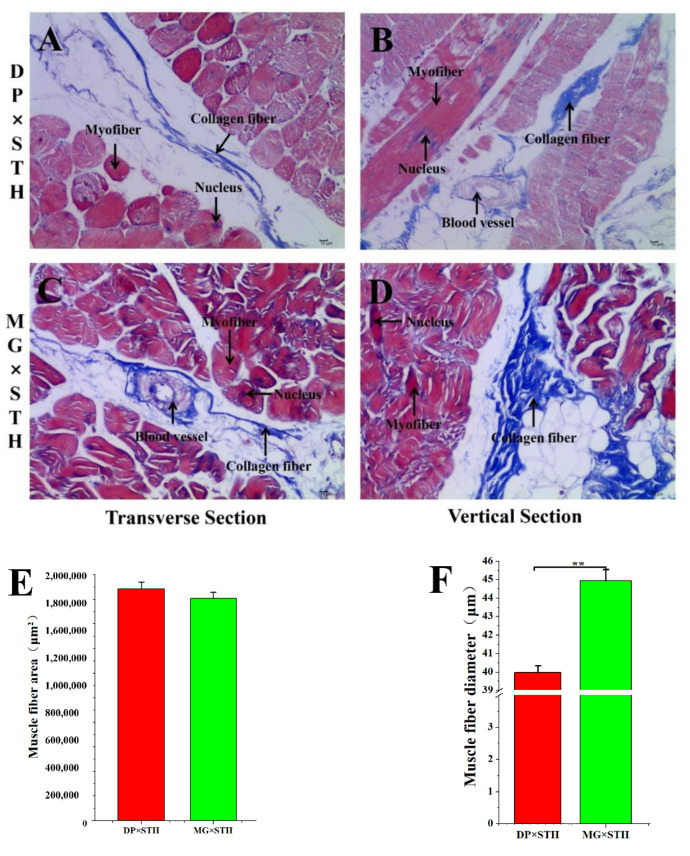
Observation of the Longissimus dorsi and muscle fiber in DP × STH and MG × STH sheep (*n* = 6). (**A**–**D**) Masson staining of Longissimus dorsi muscle. (**E**) Statistics of muscle fiber area in the visual field. (**F**) Statistics of single bundle muscle fiber diameter.

Masson staining of the *Longissimus dorsi* muscle in DP × STH and MG × STH sheep was performed to evaluate the meat quantity and muscle fibers (Figure 2). There were more muscle fibers in DP × STH sheep (Figure 2A,B) than in MG × STH sheep (Figure 2C,D); however, the collagen fibers in MG × STH sheep were more visible than those in DP × STH sheep. We calculated the muscle fiber area and diameter of the DP × STH and MG × STH sheep (Figure 2E,F); the muscle fiber area of the DP × STH sheep was larger than that of the MG × STH sheep per unit area, while the muscle fiber diameter of the DP × STH sheep was smaller than that of the MG × STH sheep. The shear force of the muscle in DP × STH sheep was smaller than that of MG × STH sheep, and the meat quality was better than that of MG × STH sheep. This concurs with the production parameters and meat quality estimates in DP × STH and MG × STH sheep.

### 3.3. Transcriptome Analysis of Longissimus dorsi Tissues

After filtering the high-throughput sequencing and raw reads quality control, an average of 1,033,646,728 and 9,847,408,899 clean reads were obtained from the *Longissimus dorsi* muscles of DP × STH and MG × STH sheep, respectively. 

**Figure 3:**
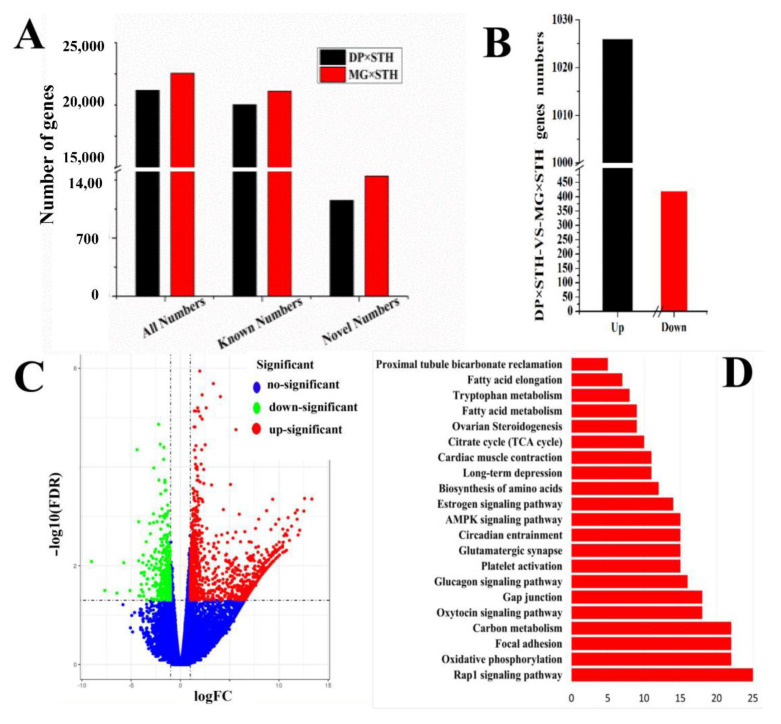

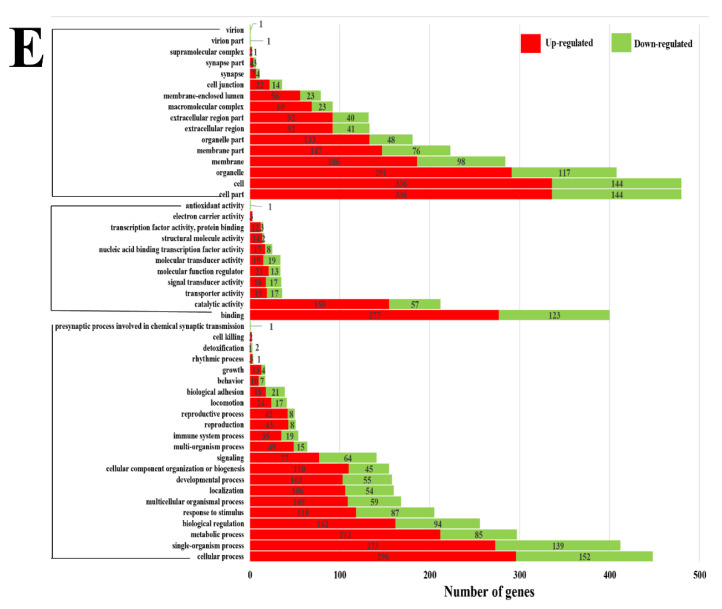
Identification identify and functional analysis of the DEGs from the Longissimus dorsi muscles in DP × STH and MG × STH sheep (*n* = 6). (**A**) Total gene numbers of transcriptome sequences in the two sheep groups. (**B**) Identity of DEGs in the two sheep. (**C**) A volcano plot of the DEGs. (**D**) The GO numbers of DEGs in cellular components, molecular function, and biological process (*p* < 0.05). (**E**) Significant pathways according to the DEGs in the two sheep (*p* < 0.05).

After screening the excess sequences, 67,220,735 and 65,731,717 sequences in the DP × STH and MG × STH sheep were mapped to the sheep genome (*Oar._v3.1*) for transcriptome analysis, respectively (Table S3). In the DP × STH sheep, 21,176 symbol genes were annotated, including 20,022 known genes and 1154 novel genes. In the MG × STH sheep population, a total of 22,556 genes were annotated, including 21,108 known genes and 1448 new genes (Figure 3A). Compared to the DP × STH group, the candidate genes with FDR < 0.05 and log_2_FC > 1 were identified as DEGs. A total of 1445 DEGs, including 1026 up-regulated and 419 down-regulated DEGs, were identified (Figure 3B,C and Table S4). A total of 49 GO terms, including 22 biological processes, 16 cellular components, and 11 molecular functions with *Q* < 0.05 were considered as the differentially expressed GO terms (Figure 3D). Pathway analysis was also performed with these DEGs, and the results suggested that a total of 21 pathways were different (*p* < 0.05 and *Q* < 0.05, Figure 3E); in particular, of the four pathways of carbon metabolism, oxidative phosphorylation, focal adhesion, and the Rap1 signaling pathway.

### 3.4. Functional Analysis of Candidate DEGs Related to Growth, Development, and Meat Quality

**Figure 4:**
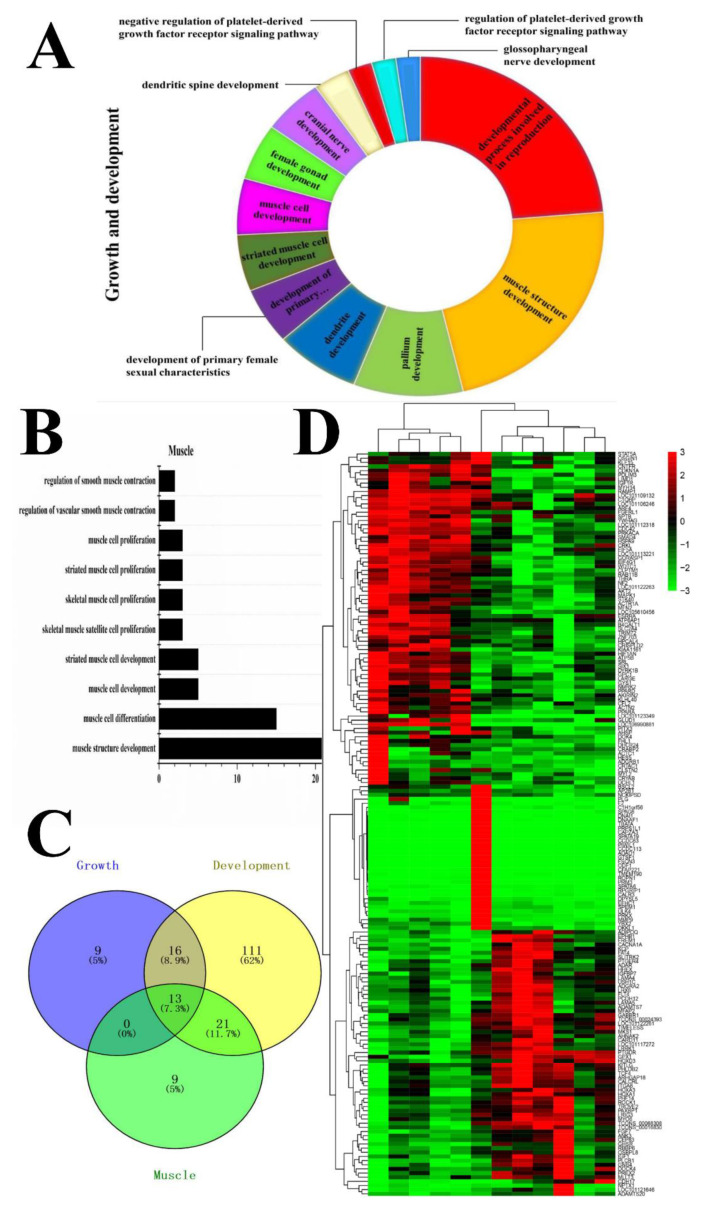
Identifying the candidate DEGs related to growth, development, and meat quality in the DP × STH and MG × STH sheep. (**A**) Identity and GO analysis of the candidate DEGs related to growth and development in the two sheep breeds. (**B**) Identity and GO analysis of the candidate DEGs related to meat quality in the two sheep groups. (**C**) The Venn diagram of DEGs related to growth, development, and meat quality. (**D**) The heat-map of the DEGs related to growth, development, and meat quality.

We selected DEGs from these GO terms that related to growth, development, and meat quality as the target genes and found significant GO items (Figure 4). A total of 13 unique GO items and 170 genes with the key words of growth and development were selected as candidate DEGs (Figure 4A). There were 10 unique GO items, including 43 genes which were highly correlated with meat quality or muscle structure (Figure 4B). For example, the GO term (GO: 0061061) for muscle structure development was chosen as the most important term that affected both growth, development, and meat quality. A Venn diagram was constructed for filtering the repeat symbol genes, and a total of 179 DEGs were identified as the candidate DEGs related to growth, development, and meat quality in the two sheep breeds (Figure 4C). Notably, 13 commonly expressed DEGs (*FGFRL1*, *SIX1*, *PLCB1*, *CRYAB*, *MYL2*, *ADIPOQ*, *GPX1*, *PPARD*, *GPC1*, *CDC42*, *LOC101106246*, *IGF1*, and *LARGE*) were found to be associated with growth, development, and meat quality. The heatmap illustrates the difference in the groups and DEGs, and most of the DEGs were significantly different (Figure 4D).

### 3.5. Validation of the Target Genes Related to Growth, Development and Meat Quality in the Longissimus dorsi 

**Figure 5:**
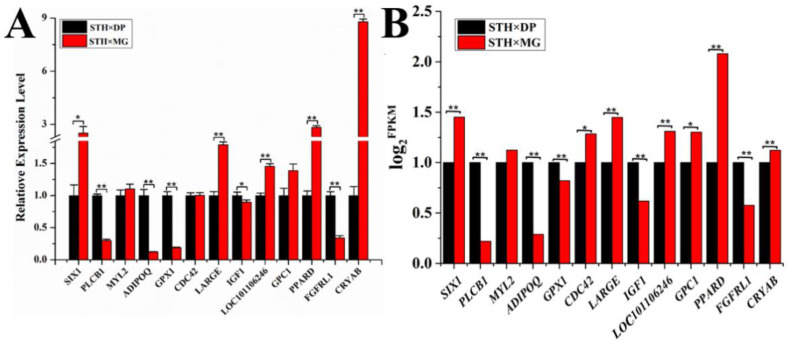
Validation of the target genes in the *Longissimus dorsi* muscle of DP × STH and MG × STH sheep. (**A**) The mRNA expression levels of thirteen co-expressed genes detected by qPCR. (**B**) Transcriptome analysis detected the mRNA expression levels of 13 co-expressed genes. * represents *p* < 0.05, ** represents *p* < 0.01.

In order to evaluate the DEGs identified in the transcriptome analysis, the 13 common DEGs were selected for mRNA validation by qPCR. The mRNA expression levels of *PLCB1*, *ADIPOQ*, *FGFRL1*, and *GPX1* genes in MG × STH sheep were down-regulated (*p* < 0.01) compared to those in DP × STH sheep. The mRNA expression levels of *LARGE*, *LOC101106246*, *PPARD*, and *CRYAB* genes were up-regulated (*p* < 0.01), especially the expression level of *CRYAB*, which was eight times higher than that of DP × STH sheep (Figure 5). The verification results of qPCR showed a similar trend to the results of transcriptome analysis (Table S5). 

### 3.6. Gene-Act-Network and Co-Expression Analysis of Targeted DEGs in Sheep Growth, Development, and Meat Quality

**Figure 6:**
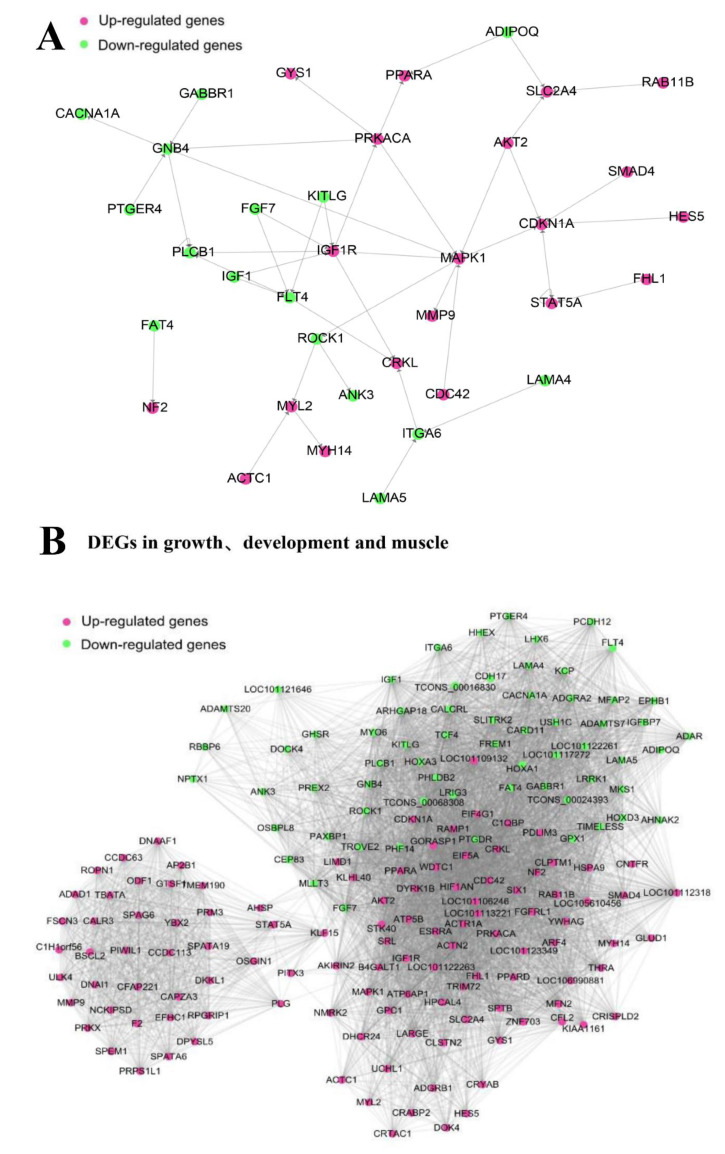
Gene-act-network and co-expression analysis of targeted DEGs in sheep growth, development, and meat quality. (**A**) The gene-act-network of the DEGs was constructed using Cytoscape software. (**B**) The co-expression analysis of targeted DEGs in sheep growth, development, and muscle.

To further explore the correlation and interaction in these DEGs, the gene-act-network and co-expression network were constructed based on 179 DEGs using Cytoscape software (Figure 6). According to the KEGG database, we first extracted the gene-act-network (Figure 6A) based on the relationships of these DEGs in expression, activation and indirect activation, phosphorylation and dephosphorylation, binding and compound, inhibition, and deletion interactions. In the gene-act-network, 36 out of 179 DEGs were found to regulate each other, especially *GNB4*, *MAPK1*, and *IGF1R*, which are co-expressed in the growth, development, and meat quality traits of sheep. Then, the co-expression network was constructed according to the Pearson correlation coefficients (*r* > 0.6, positive correlation, or *r* < 0.6, negative correlation) of the gene expression level. Of the 179 DEGs associated with growth, development, and meat quality in sheep, 13 co-expressed DEGs were found to be intrinsically related to growth, development, and meat quality (Figure 6B). 

## 4. Discussion

Sheep were among the earliest livestock raised by human beings and are also important members of current animal husbandry practices. Mutton and lamb account for a large proportion of meat consumption due to the high protein and vitamin content. As the demand for mutton and lamb and the requirements for meat quality continually increases, improving sheep performance and meat quality has become a pertinent issue. Studies have shown that crossbreeding can significantly improve sheep performance. Therefore, crossbreeding programs are constantly developing and improving [26]. Transcriptome analysis of crossbred sheep has been extensively studied. However, transcriptome analysis of two crossbred sheep breeds has not been previously reported. This experiment is significant in the study of the genetic process, productive performance, and meat quality of crossbred sheep. 

The aim of this study was to identify genes that might be associated with growth, development, and muscle by comparing two crossbred sheep. Some DEGs were related to the immune system and response in environmental adaptation (Figure 1A), indicating that the crossbred sheep retain the advantage of strong stress resistance from the Small-tailed Han sheep, which was consistent with the results of other researchers [8]. In this study, we focused on the GO-terms or DEGs associated with growth, development, and meat quality. In order to understand the main functions of these DEGs, 179 DEGs were integrated with 23 GO items, especially *FGFRL1*, *SIX1*, *PLCB1*, *CRYAB*, *MYL2*, *ADIPOQ*, *GPX1*, *PPARD*, *GPC1*, *CDC42*, *LOC101106246*, *IGF*1, and *LARGE*. In the DP × STH group, *FGFRL1*, *PLCB1*, *ADIPOQ*, and *GPX1* genes were down-regulated, while *LARGE*, *LOC101106246*, *PPARD*, and *CRYAB* genes were up-regulated, which may play a negative, regulatory role in growth and development. For example, the *PPARD* gene inhibits adipocyte differentiation at an early stage [27]. In particular, the expression level of *CRYAB* was at least eight times higher than that of DP sheep. Studies have shown that the *CRYAB* gene plays an important role in resisting both internal and external environmental stress [28]. In this study, the expression level in MG × STH sheep was much higher than that of the DP × STH sheep, indicating that MG × STH sheep had stronger adaptability and stress resistance to harsh environments than the DP × STH sheep. 

Some DEGs have been shown to play important roles in animals. For example, insulin-like growth factor (IGF), a homologous single-chain polypeptide with an insulin-like structure, is usually bound to insulin-like growth factor-binding protein (IGFBP) and is present as a complex in tissue or blood. IGF, including IGF-1, is a protein produced by the liver in response to growth hormones (GHs) secreted by the pituitary gland. IGF-1 and IGF-2, both endocrine hormones, are responsible for the growth and development of muscles and bones [29]. *ADIPOQ* is involved in the regulation of glucose and lipid metabolism and anti-apoptosis, and is directly involved in the growth and development process [30]. Glycan (GPC) is a membrane proteoglycan containing a glucose-6-phosphate isomerase (GPI) anchor. Glycans play an important role in cell development by activating multiple growth factors [31]. Fibroblast growth factor receptor-like 1(*FGFRL1*) is a novel myofibroblast growth factor receptor, which interacts with heparin and FGF ligands. The receptor (FGFRL1) is specifically required for the normal development of slow muscle fibers [32]. These studies are consistent with the results of this experiment. The SIX homeobox 1(*SIX1*) is a transcriptional regulator of a multi-gene family. Six1 can synergize with EYA transcriptional coactivator and phosphatase 2(*Eya2*) to regulate myogenesis [33]. The myosin light chain 2 (MYL2) is a member of the myosin light chain family and is a regulatory light chain. Liu’s research shows that the MYL2 gene was expressed in the *longissimus* muscle and heart, and there was almost no expression in other tissues [34]; this shows that the *MYL2* gene plays an important role in muscle development, which is consistent with the results of this test. Glutathione peroxidase 1(GPX1) also plays an important role in regulating the growth and development of muscles and has a high expression in muscle tissues [35]. In summary, through a review of the literature, it was found that the roles of eight key genes in 13 co-expressed genes in growth, development, and meat quality have been confirmed, including *PPARD* [27], *IGF1* [29], *ADIPOQ* [30], *GPC1* [31], *FGFRL1* [32], *SIX1* [33], *MYL2* [34], and *GPX1* [35]. The role of five genes (*LARGE*, *CRYAB*, *LOC10110646*, *PLCB1* and *CDC42*) in growth, development, and meat quality has not been extensively studied, and they can be used as candidate genes for further research. Transcriptome sequencing and qPCR results showed that the DEGs in the DP × STH population were different from those in MG × STH sheep. The verification results of qPCR showed the same trend as the results of transcriptome analysis, which not only verified the accuracy of transcriptome sequencing, but also indicated that these DEGs have an important regulatory effect on the growth and development of sheep and meat quality from a genetic perspective. 

The productive performance and meat quality of crossbred sheep (DP × STH and MG × STH) have been compared. Meat production (net meat growth rate, net meat weight, chest circumference) and meat quality of the DP × STH group were better than those of the MG × STH group (Table S2). The DP × STH population has better meat quality and performance; therefore, it is a better crossbreed. It is widely believed that changes in genes and proteins in muscle structure may affect phenotypes, production, and meat quality [36]. In 1972, Crouse [37] began to use histological methods to study the relationship between beef cattle muscle fibers and meat quality. The relationship between muscle fiber and muscle tenderness is the closest. Tenderness mainly refers to how the meat enters the mouth and the human experience when chewing. The better the tenderness, the better the meat tastes. Coles believes that the thinner the muscle fiber, the greater its density, and the better the meat quality [38]. Many studies believe that muscle fiber diameter and meat tenderness are negatively correlated [39]. The current study performed Masson staining and analysis in each sample. The muscle fiber area in the DP × STH group was higher than in the MG × STH group; however, the diameter of a single muscle fiber bundle in the DP × STH group was significantly smaller than in the MG × STH group. This shows that the flesh quality of DP × STH crossbreeds is better than that of MG × STH crossbreeds. This result is consistent with the performance data. In order to reveal the genetic differences between DP × STH and MG × STH sheep, a comparative transcriptome of the *Longissimus dorsi* was studied. After removing the rRNA, the ratio of the transcriptome sequence to the reference genome (*Oar_v3.1*) in each sample was above 75%, indicating that the sequencing results are highly reliable. Compared to DP × STH, a total of 1445 DEGs (including 1026 up-regulated and 419 down-regulated) were identified in MG × STH sheep. It is worth noting that in the KEGG pathways enriched by the DEGs, we found that carbon metabolism, the oxidative phosphorylation signaling pathway, and adipocytokine signaling pathway were related to the regulation of lipid metabolism, which indicates that the DP × STH and MG × STH populations with the greatest difference in lipid substitution in the Longissimus dorsi may be caused by the difference in expression levels of some related genes in the PPAR signaling pathway and the adipokine signaling pathway. The Rap1 signaling pathway exists in many important cellular processes, such as cell adhesion, cell connection, cell migration, and the control of cell proliferation and survival, therefore it has an important regulatory role in growth and development.

At present, polygene interactions are a hotspot in molecular biology. To better understand the interaction of these DEGs and the role of these DEGs in the *Longissimus dorsi* of different crossbreed sheep, we established a gene-act-network and co-expression analysis. The results showed that 24 DEGs, especially five up-regulated genes (*IGF1R*, *MAPK1*, *CDKN1A*, *MYL2*, and *PRKACA*), were found in the gene-act-network and regulated each other. In addition, we identified three hub genes (*ADIPOQ*, *GPX1*, and *MYL1*) which were also confirmed by other sheep studies [14,30] and gene behavior networks. In addition, some of the new DEGs showed a high degree of interaction between them. However, the function of these new genes is unclear. These DEGs may play an important role in sheep performance and meat quality. Further research is needed on the role of candidate genes in sheep and other livestock genetic breeding for improved productive performance.

## 5. Conclusions

In this study, we measured the performance of two crossbreed sheep, using Masson staining of muscle tissue, and using the Illumina high-throughput sequencing platform, to determine DEGs of DP × STH and MG × STH sheep, and the new transcripts of the muscle transcriptome were examined for the first time. DP × STH sheep were superior to MG × STH sheep in terms of meat quality and muscle morphology. In addition, 13 DEGs were found to play important roles in growth, development, and meat quality. The findings of this work provide valuable resources for future research on muscle development in sheep.

## Supplementary Materials

The following are available online at https://www.mdpi.com/article/10.3390/ani11061492/s1. Supplementary Table S1: qPCR primer sequences; Supplementary Table S2: Production performance and meat quality differences between the DP × STH and MG × STH sheep populations; Supplementary Table S3: Summary of transcriptomic sequence read mapping in DP × STH and MG × STH sheep populations; Supplementary Table S4: DEGs of the comparative transcriptome analysis in the DP × STH and MG × STH sheep population; Supplementary Table S5: DEGs related to growth, development and meat quality in the DP × STH and MG × STH sheep population.
